# Lyotropic Liquid Crystalline Property and Organized Structure in High Proton-Conductive Sulfonated Semialicyclic Oligoimide Thin Films

**DOI:** 10.1021/acsomega.2c06398

**Published:** 2023-02-17

**Authors:** Yuze Yao, Hayato Watanabe, Mitsuo Hara, Shusaku Nagano, Yuki Nagao

**Affiliations:** † School of Materials Science, Japan Advanced Institute of Science and Technology, 1-1 Asahidai, Nomi, Ishikawa 923-1292, Japan; ‡ Graduate School of Engineering, Nagoya University, Furo-cho, Chikusa-ku, Nagoya 464-8601, Japan; § Department of Chemistry, College of Science, Rikkyo University, 3-34-1 Nishi-ikebukuro, Toshima, Tokyo 171-8501, Japan

## Abstract

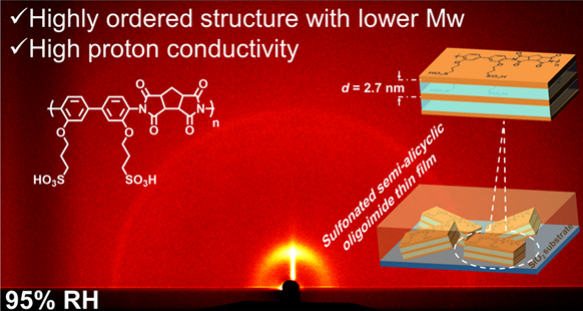

Fully aromatic sulfonated polyimides with a rigid backbone can form lamellar structures under humidified conditions, thereby facilitating the transmission of protons in ionomers. Herein, we synthesized a new sulfonated semialicyclic oligoimide composed of 1,2,3,4-cyclopentanetetracarboxylic dianhydride (CPDA) and 3,3′-bis-(sulfopropoxy)-4,4′-diaminobiphenyl to investigate the influence of molecular organized structure and proton conductivity with lower molecular weight. The weight-average molecular weight (*M*_w_) determined by gel permeation chromatography was 9300. Humidity-controlled grazing incidence X-ray scattering revealed that one scattering was observed in the out-of-plane direction and showed that the scattering position shifted to a lower angle as the humidity increased. A loosely packed lamellar structure was formed by lyotropic liquid crystalline properties. Although the ch-pack aggregation of the present oligomer was reduced by substitution to the semialicyclic CPDA from the aromatic backbone, the formation of a distinct organized structure in the oligomeric form was observed because of the linear conformational backbone. This report is the first-time observation of the lamellar structure in such a low-molecular-weight oligoimide thin film. The thin film exhibited a high conductivity of 0.2 (±0.01) S cm^–1^ under 298 K and 95% relative humidity, which is the highest value compared to the other reported sulfonated polyimide thin films with comparable molecular weight.

## Introduction

Since the great success of perfluorosulfonic acid polymer Nafion designed by DuPont in fuel cell applications, the design of proton-conducting polymers has mostly been based on the phase separation between hydrophilic and hydrophobic components. However, these materials usually do not possess well-defined long-range ordered structures, which makes it difficult to deeply discuss the relationship between structure and proton conductivity.^1−7^ After decades of research, designing polymers with efficient proton transport channels based on well-defined phase segregation is a basic principle for the development of high-performance proton-conducting materials.^5−8^

Ikkala *et al.* first observed a temperature-dependent change in proton conductivity during the order-to-disorder or order-to-order structural changes in copolymers.^9^ Kato *et al.* reported the pioneering work on the anisotropy of proton conductivity in thermotropic liquid crystal (LC) materials and demonstrated that higher proton conductivity can be obtained in channels formed by the LC.^10−12^ Rikukawa *et al.* observed anisotropic proton conductivity and swelling behavior in sulfonated poly(4-phenoxybenzoyl-1,4-phenylene)s (s-PPBPs) and proposed that this is due to the formation of LC phase by s-PPBPs in dimethyl sulfoxide (DMSO) solution.^13^ Matsui *et al.* used poly (*N*-dodecylacrylamide-*co*-acrylic acid) to obtain thin films with well-defined lamellar structures and demonstrated a huge difference in proton conductivity between in-plane (IP) and out-of-plane (OP) directions.^14^

The formation of LC structures oriented parallel to the substrate in fully aromatic and semialiphatic polyimide (PIs) films with rigid main chains was confirmed by grazing incidence X-ray scattering measurements.^15,16^ Our research group has demonstrated high IP proton conductivity in alkyl-sulfonated polyimides (ASPIs) by introducing hydrophilic sulfonated side chains into the rigid PI main chain under humidified conditions.^17^ The high IP conductivity in ASPIs is attributed to the formation of an ordered lamellar structure oriented parallel to the substrate under humidified conditions. Due to the rigid (hairy-rod) hydrophobic backbones and hygroscopic sulfonated side chains, ASPIs form certain lyotropic LC phases in a highly concentrated state by water uptake.^18,19^ These findings have brought a new perspective to investigate the relationship between structure and proton transport.^20−32^ Our previous work has systematically studied on the relationship between the organized lamellar structures and proton conductivity in ASPI thin films.^17,33−35^ ASPIs with higher molecular weight exhibit more ordered LC structures and high proton conductivity.^33,36^ Ono *et al.* reported that the increased backbone rigidity facilitates the formation of organized lamellar structures in ASPI thin films.^37^ Takakura and coauthors’ results showed that the nonlinear aliphatic ring structures in alkyl-sulfonated semialiphatic polyimides (ASSPIs) reduce the ordered structures and suppress proton conductivity.^36^ These indicate that the primary structure of the main chain is the crucial factor for the main chain rigidity (conformation) and lyotropic LC order.

In this study, we focus on ordered lyotropic lamellar structures with linear main chain conformation confirmed by density functional theory (DFT) calculation in the alkyl-sulfonated semialicyclic oligoimide composed of 1,2,3,4-cyclopentanetetracarboxylic dianhydride (CPDA) and 3,3′-bis(sulfopropoxy)-4,4′-diaminobiphenyl (BSPA). The relative humidity (RH)-controlled grazing incidence X-ray scattering (GIXRS) was used to investigate the nanostructure of the oligoimide thin film. The results show that the newly synthesized alkyl-sulfonated semialicyclic oligoimide forms a lamellar structure. The proton conductivity of the alkyl-sulfonated semialicyclic oligoimide is as high as 0.2 (±0.01) S cm^–1^ (298 K, 95% RH), which is the highest value among the reported ASPI thin films with comparable molecular weight. Furthermore, we summarized the effects of molecular weight as well as main chain conformation on the lyotropic LC properties, providing new insights for the molecular design of high proton-conducting materials.

## Experimental Section

### Materials

BSPA was synthesized according to the previous reports.^17^ Triethylamine (TEA) was used as received from Kanto Chemical Co. Inc., Japan. Hydrochloric acid, m-cresol, acetic acid, acetic anhydride, methanol, and acetone were obtained from Fujifilm Wako Pure Chemical Corp., Japan. CPDA was purchased from Tokyo Chemical Industry Co. Ltd., Japan.

### Synthesis of BSPA–CPDA

**Scheme 1:**
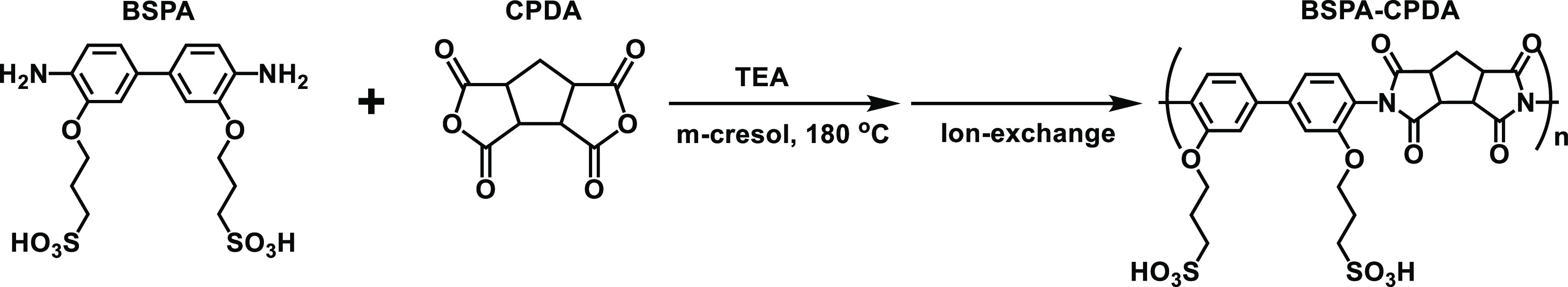
Synthesis of BSPA–CPDA

As shown in Scheme 1, the oligoimide (BSPA–CPDA) was newly synthesized by a “one-pot” method. 1 mmol BSPA (0.46 g), 1 mmol CPDA (0.21 g), 10 mL of m-cresol, and 600 μL of TEA were added to a 50 mL three-necked flask with a magnetic stirrer under an argon atmosphere. The mixture was stirred at 80 °C for 2 h. Then, the temperature was raised to 180 °C to continue the reaction. After reaction for 20 h, the polymerized mixture was cooled to room temperature and poured into fresh cold acetone to obtain a white precipitate. The precipitate was washed several times with acetone, separated by centrifugation, and then dried under vacuum overnight. After the obtained precipitate was subjected to an ion exchange operation using Amberlyst 31WET (Organo Corporation), the final product BSPA–CPDA was obtained. The chemical structure of BSPA–CPDA was confirmed by ^1^H NMR spectra using a spectrometer (400 MHz, Bruker AVANCE III; Bruker Analytik GmbH). The deuterated DMSO with tetramethyl silane was used as the solvent. The molecular weight of the final product was measured by gel permeation chromatography (GPC).

### Thin-Film Preparation

Before thin film deposition, Si, SiO_2_ substrates (E&M Co. Ltd.), and SiO_2_-coated 9 MHz quartz crystals (Seiko EG&G Co. Ltd.) were washed with 2-propanol. Before thin-film deposition, 10 s plasma treatment (Cute-MP; Femto Science, Korea) was carried out to improve the hydrophilic properties of the substrate surface. Thin films were prepared on these substrates from 6.5 wt % BSPA–CPDA solution in a mixture of Milli-Q water and tetrahydrofuran with a weight ratio of 1:1. The spin-coating method was carried out by a spin-coater (ACT-200D; Active Co. Ltd.). The thin-film thickness was controlled to around 500 nm. The thickness of the thin films was measured by a white light interference microscope (BW-S506; Nikon Corp.)

### Water Uptake

Water uptake was measured using an *in situ* quartz crystal microbalance (QCM) system. QCM substrates were connected to an oscillation circuit with a DC power supply and a frequency counter (53131A; Agilent Technologies Japan Ltd.). The QCM substrate was placed in an in-house constructed humidity chamber with a high-resolution RH sensor. Various RHs in the experiment were produced using dry N_2_ gas and humidified streams applied by a humidity controller (BEL Flow; BEL Japan Inc.). When the QCM substrate reaches equilibrium under a certain humidity condition, its frequency fluctuates within a certain range. The average value of the frequencies at this time was used as the frequency of the QCM substrate. The mass of the dried thin film under dry N_2_ atmosphere was calculated by measuring the change in frequency before and after spin coating of the QCM substrate through the Sauerbrey equation
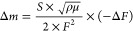
1where *S* represents the electrode surface area, *ρ* and *μ* denote the quartz density and quartz shear modulus, respectively, and *F* stands for the fundamental frequency of the QCM substrate.

The water content *λ*, which represents the number of water molecules per sulfonic acid group, was calculated using the equation shown below
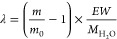
2*m* represents the mass of the thin film under each humidity, *m*_0_ represents the mass of the thin film at 0% RH,  stands for the molecular weight of water molecules, and *EW* expresses the equivalent weight of BSPA–CPDA.

### *In Situ* FTIR

The dissociation state of the sulfonic acid group was investigated by RH *in situ* Fourier transform infrared (FTIR) measurements. The thin-film sample made on silicon wafer was placed in homemade chambers. CaF_2_ windows were used in the humidity-controlled cell. An FTIR spectrometer (Nicolet 6700; Thermo Fisher Scientific Inc.) equipped with a deuterium triglycine sulfate detector was used for transmission *in situ* FTIR measurements. The RH change was controlled within the range of 0–95% using a humidity generator (me-40DP-2PW; Microequipment). For these experiments, the RH when running dry nitrogen was defined as 0%.

### GIXRS

RH-controlled *in situ* GIXRS was measured by a FR-E X-ray diffractometer equipped with R-AXIS IV two-dimensional (2D) detector (FR-E; Rigaku Corp). The thin-film sample was placed in a humidity-controlled cell with X-ray transparent polyester film (Lumirror) windows. The humidity in the cell was controlled using the humidity generator (me-40DP series). X-rays with a wavelength of 0.1542 nm were generated through Cu *K*α radiation with a beam size of approximately ϕ300 μm. The camera length was 300 mm, and the incidence angle was set in the range of 0.20–0.22°. The integrated regions in 1D IP and OP patterns were taken between −0.5 to +0.5° from the center (0°) as 2*θ* (for IP) or *α* (for OP), respectively.

### Molecular Structure Simulation

The optimized molecular structures were calculated by Material Studio 2020. The calculations were done based on DFT using a DMol3 module. Generalized gradient approximation functional with the Perdew–Burke–Ernzerhof type was used to model the exchange and correlation interactions. Convergence threshold for the maximum force and maximum displacement for normal geometry optimization were set, respectively, to 0.002 Ha Å^–1^ and 0.005 Å.

### Proton Conductivity

In order to measure the proton conductivity of the thin film in the direction parallel to a substrate surface, a frequency response analyzer and a high-frequency dielectric interface (SI1260 and SI1296; Solartron Analytical) were used. Gold paste was used to make electrodes for conductivity measurements. A humidity- and temperature-controlled chamber (SH-221; Espec Corp.) was used to control the humidity and temperature during the experiment. The data of the impedance were collected by application of an alternating potential of 50 mV over frequencies ranging from 10 MHz to 1 Hz. The collected impedance values (*R*) were used to calculate the conductivity of the thin film directly by using the formula
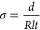
3where *t* stands for the thin film thickness, *l* represents the contact electrode length, and *d* is the space between the gold electrodes.

## Results and Discussion

### Characterization of BSPA–CPDA

The chemical structure of BSPA–CPDA was confirmed by ^1^H NMR spectra (Figure S1). The degree of sulfonation and ion exchange capacity for the final product were more than 95% and 3.0 meq g^–1^, respectively. The FTIR spectra were measured in the range of 400–4000 cm^–1^, as shown in Figure S2. The peaks observed at 3415 and 2944 cm^–1^ are attributed to the stretching vibration of N–H and C–H bonds, respectively. The absorption peaks of ν_s_ (C=O), ν_as_ (C=O), and ν (C–N) were observed at 1778, 1706, and 1383 cm^–1^, respectively. The peak observed at 1502 cm^–1^ is attributed to the stretching vibration of the C–C bond. The asymmetric stretching vibration and symmetric stretching vibration peaks of the sulfonic acid groups were observed at 1249 and 1193 cm^–1^, respectively. The number-average molecular weight (*M*_n_) and weight-average molecular weight (*M*_w_) were 4300 and 9300, respectively (Figure S3 and Table S1). The calculated average degree of polymerization was 14. Compared to the PIs with a cyclohexane structure reported by Takakura and coauthors (*M*_w_ = 25,000),^33^ the molecular weight of oligoimide in this study was much lower.

### Water Uptake

**Figure 1:**
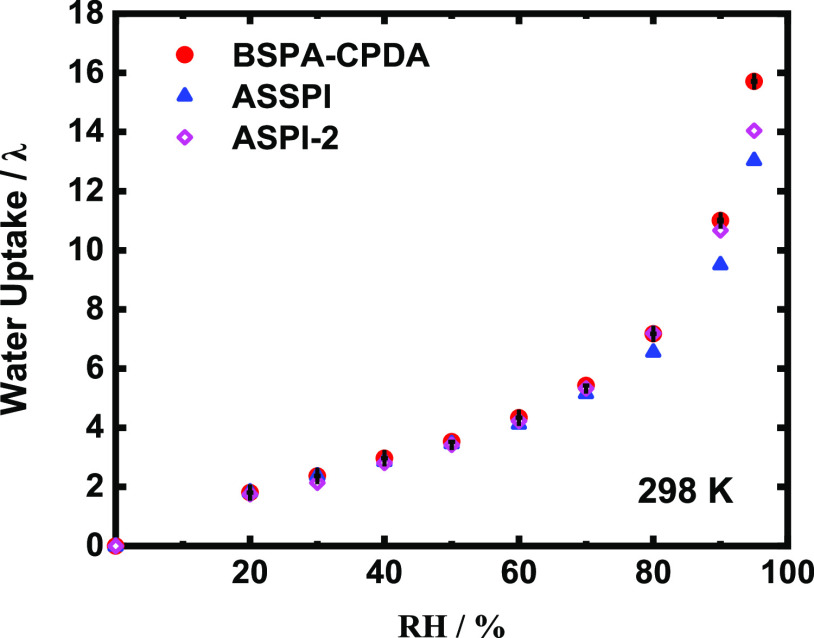
Water uptake of BSPA–CPDA (with error bars), ASPI-2,^33^ and ASSPI^36^ thin films as a function of RH at 298 K.

Water uptake is an important factor affecting the proton conductivity because water acts as a carrier to facilitate the transport of protons in thin films.^6^Figure 1 shows the RH-dependent water uptake of the BSPA–CPDA thin film. For comparison, the water uptake data for ASSPI (consisting of 1,2,4,5-cyclohexanetetracarboxylic dianhydride and BSPA, Figure S4a)^36^ and ASPI-2 (consisting of pyromellitic dianhydride and BSPA, Figure S4b)^33^ thin films with comparable molecular weights are also plotted in the same figure. The adsorption isotherm of water molecules showed a tendency similar to the adsorption isotherm of nonporous multimolecular adsorption. There was considered to be a change in the type of adsorbed water around the sulfonic acid groups.^38,39^ The adsorption behaviors of BSPA–CPDA, ASPI-2, and ASSPI thin films were similar with respect to RH. The water uptake value gradually increased concomitantly with increasing RH. It is apparent that the water uptake value of all thin films was the same under low humidity, but the BSPA–CPDA thin film showed a higher water uptake value (*λ* = 16) than ASSPI (*λ* = 13) and ASPI-2 (*λ* = 14) thin films at 95% RH.

### *In Situ* FTIR

**Figure 2:**
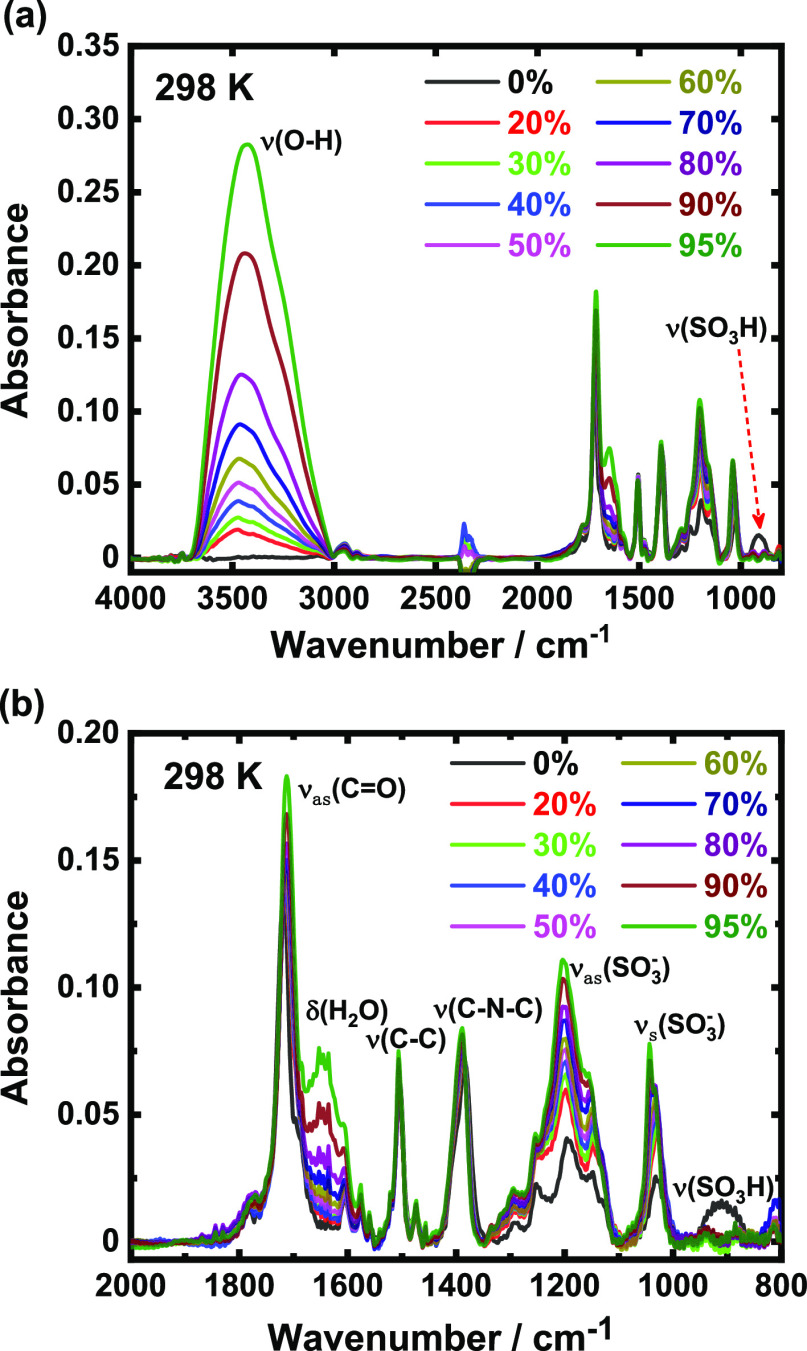
RH-dependent FTIR spectra of the BSPA-CPDA thin film for (a) 4000–800 cm^–1^ and (b) 2000–800 cm^–1^.

To evaluate the dissociation behavior of protons at sulfonic acid groups, RH-controlled *in situ* FTIR measurements were performed on the BSPA–CPDA thin film. The spectra of the BSPA–CPDA thin film under humidification are shown in Figure 2a. The broad absorption band around 3420 cm^–1^ is attributed to the OH stretching vibration mode of water molecules under humidification.^40,41^ The absorbance of this band increased with increasing RH, indicating that water molecules were adsorbed onto the thin film under humidified conditions. Specifically, at 0% RH, the absorption band of water molecules could not be observed; meanwhile, the band observed at 902 cm^–1^ was attributed to the stretching vibration mode of the S–O bond of protonated sulfonic acid groups.^42^ This band at 902 cm^–1^ disappeared completely with increasing RH.

Subsequently, the molecular states of the sulfonic acid group of the BSPA–CPDA thin film were analyzed. As shown in Figure 2b, the absorption band attributed to the O=S=O symmetric stretching vibration (ν_s_ (SO_3_^–^)) of deprotonated sulfonic acid was observed at 1030–1040 cm^–1^. It is noteworthy that some sulfonic acid groups are deprotonated under 0% RH. The peak area of ν_s_ (SO_3_^–^) under different RH conditions was recorded as *S*_*x*_(SO_3_^–^), and the peak area of ν_s_ (SO_3_^–^) at 0% RH was recorded as *S*_0_(SO_3_^–^). The proton dissociation (PD/%) rate of the sulfonic acid group under each humidity condition is defined by the following equation
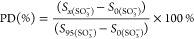
4

**Figure 3:**
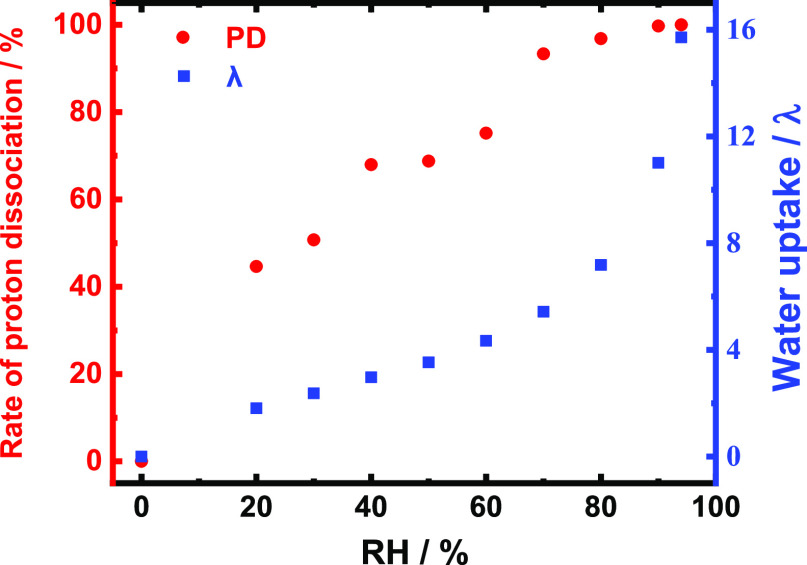
RH-dependent rate of proton dissociation from ν_s_ (SO_3_^–^) and water uptake for the BSPA–CPDA thin film.

The obtained PD value and water uptake value are shown as a function of RH in Figure 3. Below 70% RH, the PD value increased to 90% rapidly with a small amount of water uptake, which indicates that even less water adsorption can cause rapid deprotonation of sulfonic acid groups. When the RH further increased from 70 to 95%, the PD value only increased by 10% and saturated. The sulfonic acid groups are considered to be completely deprotonated at 95% RH.

The change in PD behavior is related to the type of water molecules adsorbed around the sulfonic acid groups. Zhao *et al.* reported that the adsorbed water molecules around sulfonic acid groups in the Nafion membrane can be divided into two types: one type is caused by forming a primary hydration shell by strong-binding water molecules with acid groups under the *λ* region less than 4; another type is caused by the hydration of more excess water molecules under the *λ* region more than 4.^43^ In our study, the slope of the water uptake changed quickly at around *λ* = 5 as 70% RH, indicating a change from the water strongly bound to sulfonic acid groups to excessive bulk water.

### *In Situ* GIXRS

**Figure 4:**
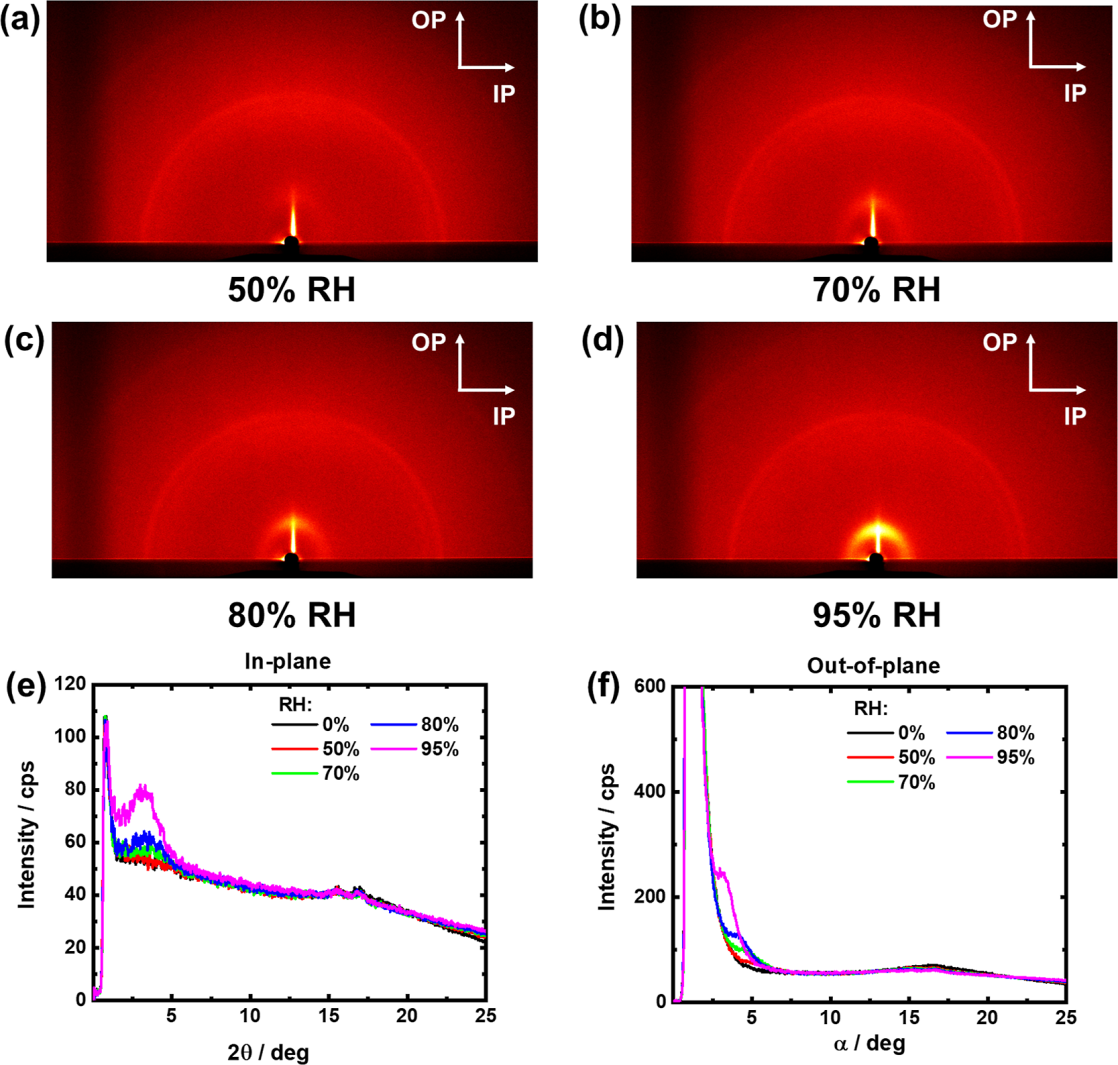
GIXRS results of the BSPA–CPDA thin film. 2D GIXRS profiles at (a) 50% RH, (b) 70% RH, (c) 80% RH, and (d) 95% RH. RH-dependent 1D profiles in the (e) IP and (f) OP directions, respectively. Those scattering peaks at 2*θ* = 15.5 and 16.8° originate from the Lumirror window of the humidity-controlled cell.

GIXRS is a powerful tool for detecting molecular packings and orderings in organized thin films.^44,45^ In order to investigate the effects of the oligomeric semialicyclic main chain on the lyotropic organized structure, RH-dependent *in situ* GIXRS measurements were performed on the BSPA–CPDA thin film. The 2D scattering images are shown in Figure 4a–d and 1D GIXRS profiles in the IP and OP directions are shown in Figures 4e,f. One scattering peak in the OP direction was observed, indicating that the ordered structure was formed perpendicular to the substrate surface. According to our previous reports, the hydrophobic backbone of PIs aligned along the IP direction parallel to the substrate, meanwhile the hydrophilic side chain with sulfonic acid groups oriented in the OP direction to form a lamellar structure by lyotropic LC properties.^8,36,37^ In the present study, as the RH increased, the intensity of the scattering peak increased in the OP direction, and the peak position gradually moved from 2*θ* = 4.7° (50% RH) to 3.0° (95% RH). This trend of structural change is the same as that presented in previous reports, indicating that a loosely packed lamellar structure and degree of molecular ordering were enhanced by the lyotropic LC properties.^8,36,37^ In the IP profiles, RH-dependent scattering peaks were observed in the small-angle region. This scattering peak can be attributed to the origin from the broad OP scattering as shown in Figure 4b–d. These results indicate that, as the RH increases, the loosely packed lamellar was organized and expanded to the OP direction with an increase in the degree of structural order. Polarized optical microscopy was used to confirm the lyotropic LC-like domain morphologies. However, due to the rapid loss of adsorbed water molecules under ambient temperature and humidity conditions, birefringence was not observed as shown in Figure S5. This is consistent with the absence of scattering peaks in the GIXRS results at low humidity conditions.

Ando *et al.* have investigated the backbone aggregation of PIs with both aromatic and semialiphatic structures through grazing-incidence wide-angle X-ray scattering measurement.^16^ The fully aromatic PI with the rod-like molecular structure and high planarity forms a smectic LC ordered structure. Our previous fully aromatic ASPI thin films with alkyl-sulfonated side chains also showed similar IP and OP scatterings, which can be attributed to the periodic unit length and a ch-pack aggregation of the PI backbone, respectively.^17,33,37^ In the case of ASSPI, no scattering representing the periodic unit length was observed in the IP direction, and the scattering representing ch-pack aggregation was observed in the OP direction.^36^ However, only one isotropic (arc) scattering was observed in the GIXRS results of low-molecular-weight ASSPI, indicating that even under humidified conditions, low-molecular-weight ASSPI only exhibits a weak randomly oriented lamellar structure due to the semialicyclic main chain. In the present study, the GIXRS results are similar to those of the ASSPI case. A weak RH-independent scattering was observed at *α* = 16.5°, representing the ch-pack aggregation of the BSPA–CPDA backbone in the OP direction. Although the ch-pack interaction of the present oligomer is reduced by the substitution of semialicyclic CPDA, we were able to observe the formation of a distinct organized lamellar structure in the OP direction under high-RH conditions.

**Figure 5:**
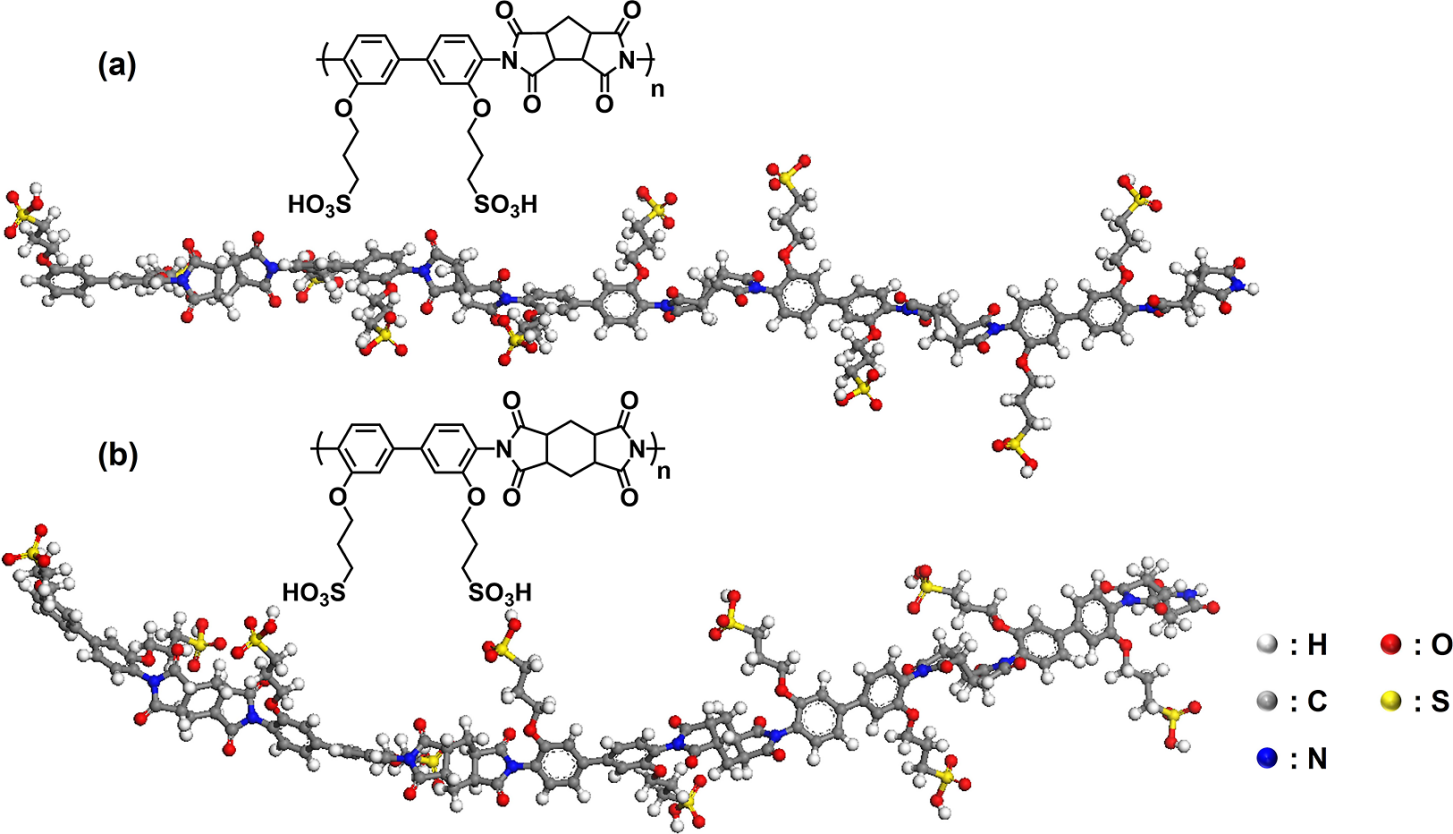
Optimized structures of (a) five repeating units of BSPA–CPDA with rigid backbone and (b) five repeating units of ASSPI with bending backbone.

To understand the reason for observing the lamellar structure in oligomeric BSPA–CPDA, we tried to investigate the structural model. For comparison, oligomeric ASSPI units that only show a weak lamellar structure were also considered. Figure 5 depicts the optimized oligomeric structures of five repeating units for BSPA–CPDA and previous ASSPI by DFT calculation. The main chain of BSPA–CPDA showed a more linear conformation than that of ASSPI. Therefore, even with low molecular weight, BSPA–CPDA thin film with a more rigid backbone can exhibit a well-ordered lamellar structure driven by strong lyotropic LC properties.

**Figure 6:**
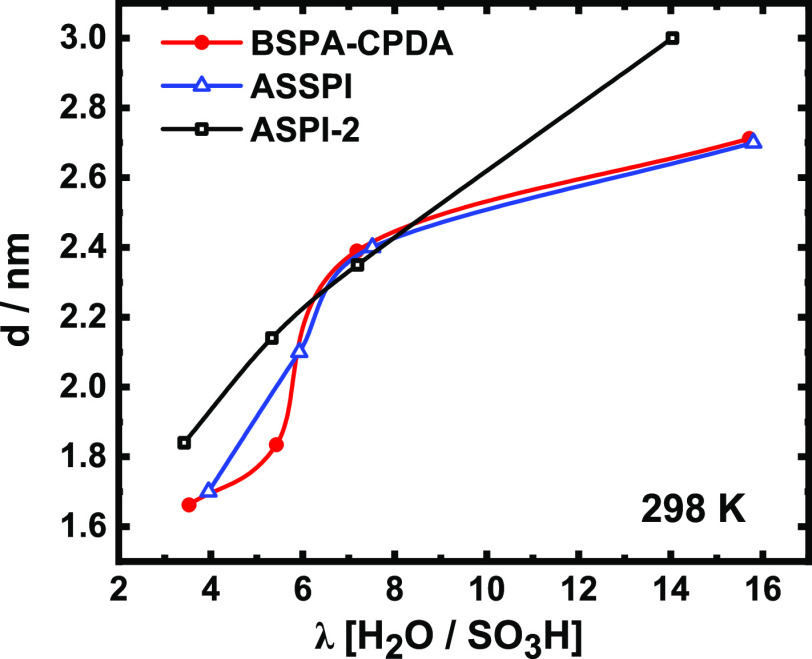
Humidity-dependent layer distance of BSPA–CPDA, ASPI-2,^37^ and ASSPI^36^ thin films.

The layer distances (*d*) of the lamellar structure under different humidity conditions are calculable according to the following equation
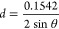
5where *θ* is the angle of scattering peak appearing at different RH values and 0.1542 (nm) is the wavelength of the X-rays generated through Cu *K*α radiation. Figure 6 shows the calculated *d* value of the BSPA–CPDA thin film as a function of water uptake. For comparison, the *d* values of the other two reported ASPI thin films are shown.^36,37^ The layer distance of the fully aromatic ASPI-2 thin film changes linearly, showing a maximum *d* value of 3.0 nm at *λ* = 14. The layer distance of semialicyclic sulfonated oligo-BSPA–CPDA and ASSPI thin films varied nonlinearly with a relatively lower maximum *d* value of 2.7 nm at *λ* = 16. Nonlinear expansion of the interlamellar distance with respect to the water uptake value has not been observed in the fully aromatic PI. This is considered to be a feature of thin films having a semialicyclic backbone, although the mechanism is not yet determined. In addition, in fully aromatic PI thin films, the length of the side chain determined the interlamellar distance. However, this tendency clearly differs in the semialicyclic oligoimide thin film having an alkyl side chain with the same length. Because the semialicyclic oligoimide thin film has a small interlamellar distance, the proton concentration per unit volume is higher than that of the fully aromatic PI thin films. Therefore, high proton conductivity can be expected.

### Proton Conductivity

**Figure 7:**
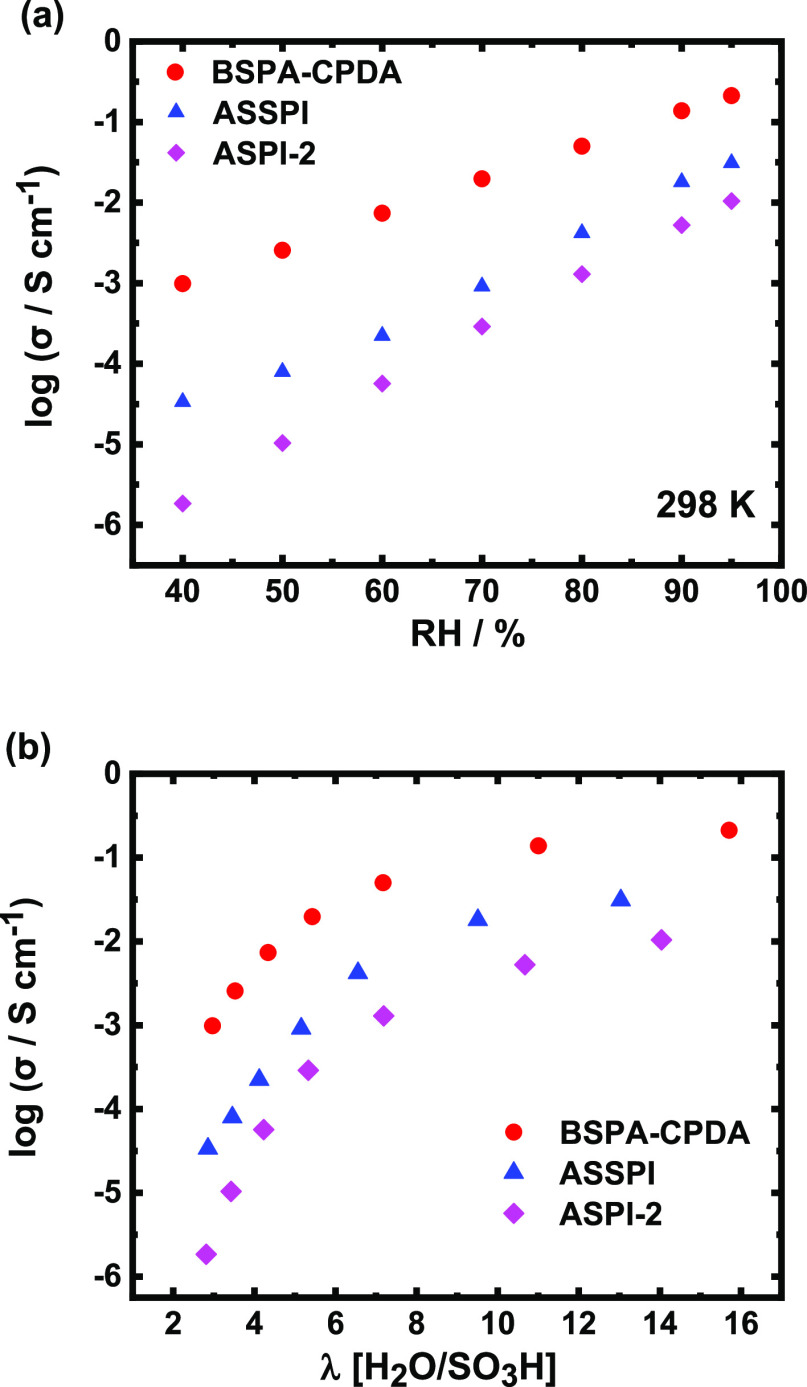
Proton conductivity of BSPA–CPDA, ASPI-2,^33^ and ASSPI^36^ thin films with comparable molecular weight (a) as a function of RH and (b) as a function of water uptake (*λ*) at 298 K.

The proton conductivity for the BSPA–CPDA thin film is shown in Figure 7a as a function of humidity at 298 K. The proton conductivity increased with increasing RH, which can be observed in typical proton-conducting polymers. The maximum proton conductivity reached 0.2 (±0.01) S cm^–1^ (at 298 K, 95% RH), which is the highest value among the reported sulfonated PI thin films with comparable molecular weight.^33,36^ It is noteworthy that these ASPI thin films show molecular weight dependence of the proton conductivity.^33^ Therefore, we compared the proton conductivity of thin films which had a similar low molecular weight. Figure 7b shows the proton conductivity of BSPA–CPDA, ASPI-2, and ASSPI thin films as a function of the water uptake value. Increasing the water uptake value in the case of low RH increased the proton conductivity of all thin films significantly. At the same water uptake, the BSPA–CPDA thin film showed the highest proton conductivity. This highest proton conductivity can be attributed to the formation of organized lamellar structure due to the strong lyotropic LC properties in semialicyclic oligoimide with a linear backbone.

### Comparison with Other ASPIs

Table 1 summarizes the structural features, molecular weight, LC features, and proton conductivity of the BSPA–CPDA and reported ASPI thin films.^33,36,37^ The ASPI-1 (consisting of 1,4,5,8-naphthalenetetracarboxylic dianhydride and BSPA, Figure S4c) and ASPI-2 have a fully aromatic backbone, and the ASSPI^33^ and the present BSPA–CPDA possess a semialicyclic backbone. In all alkyl-sulfonated PI thin films, a lyotropic lamellar ordering with phase separation of the hydrophobic main chain and hydrophilic side chain layers is observed under humidification. Moreover, the fully aromatic ASPI-1 and ASPI-2 thin films exhibit smectic ordering within the lamellar layer because of the lyotropic LC properties by the rigid linear main chain.^33,37^ Reportedly, the degree of molecular ordering deteriorates when the molecular weight becomes small.^33^ On the other hand, the previous ASSPI thin film with a semialicyclic backbone does not exhibit scattering corresponding to the main chain smectic order in the intralamellar plane.^36^ This indicates that the alicyclic structure weakens the aggregate of the main chains, and the positional order of the main chains within the lamellar layer is lost (nematic-like structure). Therefore, the scattering of the lyotropic lamellar structures is also weak and less ordered compared to the fully aromatic PIs. The lamellar ordering decreased considerably in the low-molecular-weight ASSPI (*M*_w_ = 25,000).

**Table 1:** Structural Features, Molecular Weight, LC Features, and Proton Conductivities of the Present BSPA–CPDA and Reported ASPI Thin Films with Thickness of ∼500 nm

materials	main chain backbone	main chain conformation	molecular weight (*M*_w_)	lyotropic LC structure	main chain LC structure	*σ*^a^ (S cm^–1^)
BSPA–CPDA	semialicyclic	linear	9300	lamellar	nematic-like	0.20
ASPI-1^37^	aromatic	linear	490000	lamellar	smectic	0.18
ASPI-2^33^	aromatic	linear	13000	weak lamellar		0.01
			260000	lamellar	smectic	0.26
ASSPI^36^	semialicyclic	nonlinear	25000	weak lamellar		0.03
			40000	lamellar	nematic-like	0.15

a Proton conductivity measured at 298 K and 95% RH.

In the present BSPA–CPDA with a semialicyclic backbone, the scattering corresponding to the lyotropic lamellar organized structure was observed, but no positional order of the main chains was observed. Even for the oligomer level of the molecular weight (*M*_w_ = 9,300), BSPA–CPDA obviously exhibits lyotropic lamellar scattering, indicating highly molecular ordering compared to ASSPI. Comparing the two semialicyclic polymers, the present BSPA–CPDA adopts a more linear conformation according to the DFT results (Figure 5). Therefore, BSPA–CPDA with a more linear main chain exhibits a higher-ordered lamellar structure than ASSPI. Proton conductivity decreased greatly with decreasing molecular weight in previous ASPI and ASSPI. However, the BSPA–CPDA oligomer exhibits high proton conductivity, comparable to ASPIs with higher molecular weight. The ordered lamellar structure driven by the linear conformation of the semialicyclic and rigid backbone structure can enhance the proton conductivity. This is the first demonstration of the high proton conductivity by the lamellar structure with a semialicyclic backbone in such a low-molecular-weight oligoimide thin film.

## Conclusions

To date, sulfonated semialicyclic PI thin films have not been reported to form a lamellar structure because of weak rigidity of the main chain and low molecular weight. In this study, we investigated whether lyotropic LC properties can drive a lamellar structure by improved linear conformation of the semialicyclic main chain, even with a low molecular weight. To investigate this hypothesis, a novel sulfonated semialicyclic oligoimide, BSPA–CPDA, with a cyclopentane structure was newly synthesized, employing a more linear conformational main chain than previously reported. The water uptake of the BSPA–CPDA thin film showed a trend similar to that of other reported ASPIs; the results of *in situ* FTIR showed that when *λ* = 5 at 70% RH, the adsorbed water around the sulfonic acid group changes from bound water to bulk water. The GIXRS results of the BSPA–CPDA thin film showed that the scattering peak of the loosely packed lamellar structure driven by lyotropic LC properties was observed under high-humidity conditions, indicating a change from disordered to ordered aggregated structure. As the humidity increased, the lamellar structure was strengthened and the lamellar distance increased. The proton conductivity of the BSPA–CPDA thin film increased with increasing humidity and achieved a value of 0.2 (±0.01) S cm^–1^ at 298 K and 95% RH. Compared with the other reported ASPIs with lower molecular weight, the BSPA–CPDA thin film has the highest proton conductivity value. This high conductivity is attributed to the formation of the lamellar structure to facilitate proton transport driven by the nature of lyotropic LC properties. We concluded that the sulfonated semialicyclic oligoimide thin films with more linear conformational main chain can form an organized lamellar structure because of the strong lyotropic LC properties.

## Supporting Information Available

Characterization of sulfonated semialicyclic oligoimide, ^1^H NMR spectra, FTIR spectra, GPC measurement, chemical structure of ASPI-1, ASPI-2, and ASSPI, and polarized optical microscope images (PDF)
