# Supplementary material for: Lyotropic Liquid Crystalline Property and Organized Structure in High Proton-Conductive Sulfonated Semialicyclic Oligoimide Thin Films
Source: ACS Omega. 2023 Feb 17;8(8):7470–8. doi: 10.1021/acsomega.2c06398 (PMC9979332; doi:10.1021/acsomega.2c06398)
Supplement: Supplementary file 1 — ao2c06398_si_001.pdf [file ao2c06398_si_001.pdf]

Supporting Information for

Lyotropic Liquid Crystalline Property and Organized Structure in High Proton-Conductive Sulfonated Semi-Alicyclic Oligoimide Thin Films

Yuze Yao † *Hayato Watanabe* ‡ *Mitsuo Hara* ‡ *Shusaku Nagano* § *Yuki Nagao* †*

†School of Materials Science, Japan Advanced Institute of Science and Technology, 1-1 Asahidai, Nomi, Ishikawa 923-1292, Japan.

‡Graduate School of Engineering, Nagoya University, Furo-cho, Chikusa-ku, Nagoya, 464-8601, Japan.

§Department of Chemistry, College of Science, Rikkyo University, 3-34-1 Nishi-ikebukuro, Toshima, Tokyo 171-8501, Japan.

Table of Contents

1. ^{1}H nuclear magnetic resonance (^{1}H NMR) study S2

2. FTIR study S3

3. Gel permeation chromatography (GPC) study S4

4. Chemical structure of ASPI-2 and ASSPI S5

5. Polarized Optical Microscopy (POM) S6

References S7

1. ^{1}H nuclear magnetic resonance (^{1}H NMR) study

The ^{1}H NMR results of as synthesized monomer 3,3'-bis(sulfopropoxy)-4,4'-diaminobiphenyl (BSPA) and oligoimide (BSPA-CPDA) are shown in Figure S1. In the results, the peak appearing around 2.5 ppm was attributed to DMSO-d6. In the NMR result of BSPA, the broad peak appeared at 3.3-3.5 ppm is attributed to the water. Similarly, the water peak was observed at 5.0–5.8 ppm in the NMR results of BSPA-CPDA. The peak appeared at δ = 6.9–7.9 ppm corresponding to the hydrogen atom of benzene rings, and the peaks appeared at 2.1–4.2 ppm are attributed to the protons of the alkoxy chains for both BSPA and BSPA-CPDA. The peak at δ = 1.1 ppm is attributed to the remaining triethylamine (TEA). The integral value decreased from 18 (before ion exchange) to 0.26 (after ion exchange), indicating that more than 98% of TEA was replaced by proton. In the results of BSPA-CPDA, peaks from the possible by-product amide or carboxyl group were not observed, which proved that the imidization reaction was complete. Integration of proton peaks in ^{1}H NMR spectra were in good agreement with the number of protons in both the backbone and side chains.

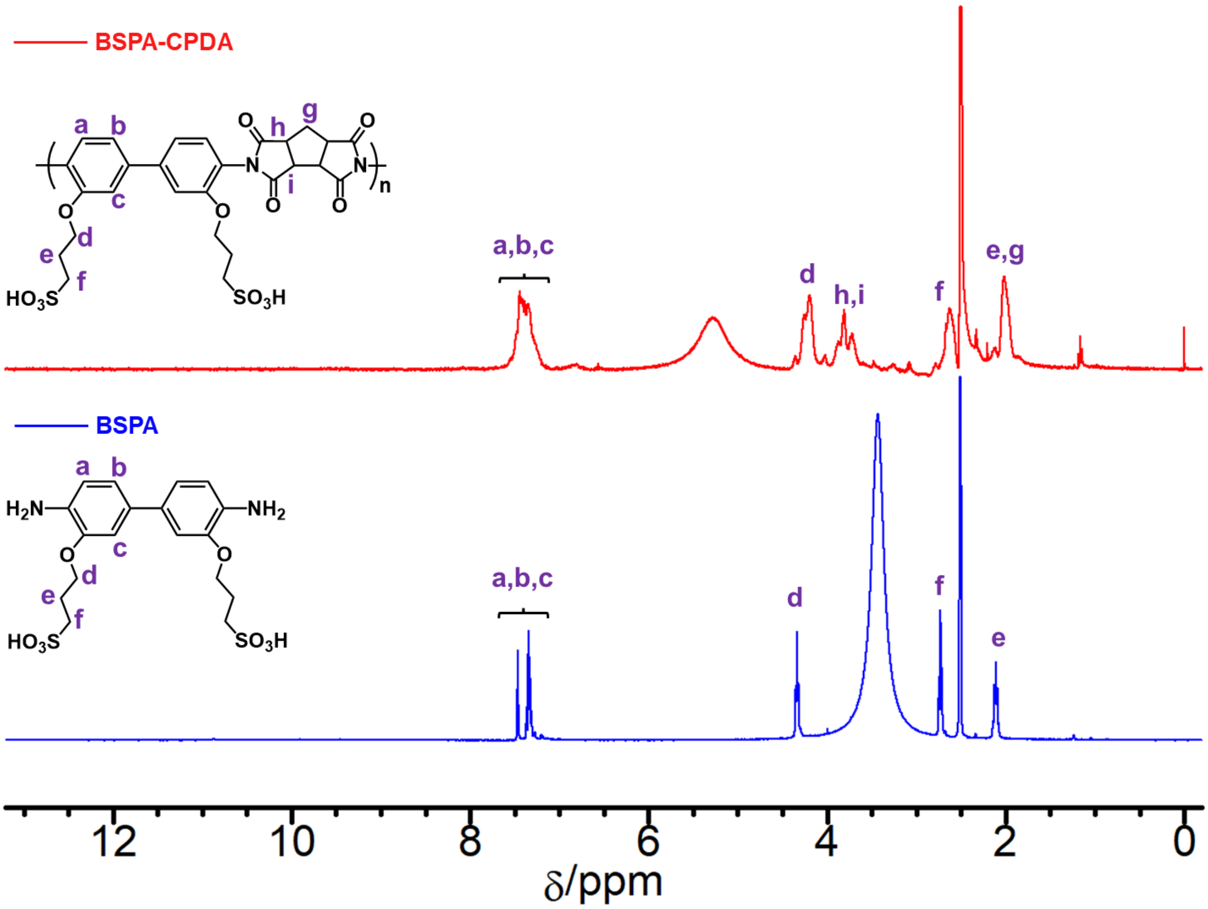

Figure S1. ^{1}H NMR (DMSO-d6) spectra of BSPA and BSPA-CPDA.

2. FTIR study

FTIR spectra of BSPA and BSPA-CPDA are shown in Figure S2. The peak observed at 3415 cm^{-1} and 2944 cm^{-1} are attributed to stretching vibration of N–H and C–H bonds, respectively. The absorption peaks of ν_s (C=O), ν_{as} (C=O) and ν (C–N) were observed at 1778 cm^{-1}, 1706 cm^{-1} and 1383 cm^{-1}, respectively. The peak observed at 1502 cm^{-1} is attributed to the stretching vibration of C–C bond. The asymmetric stretching vibration peak and symmetric stretching vibration peak of the sulfonic acid groups were observed at 1249 cm^{-1} and 1193 cm^{-1}, respectively.

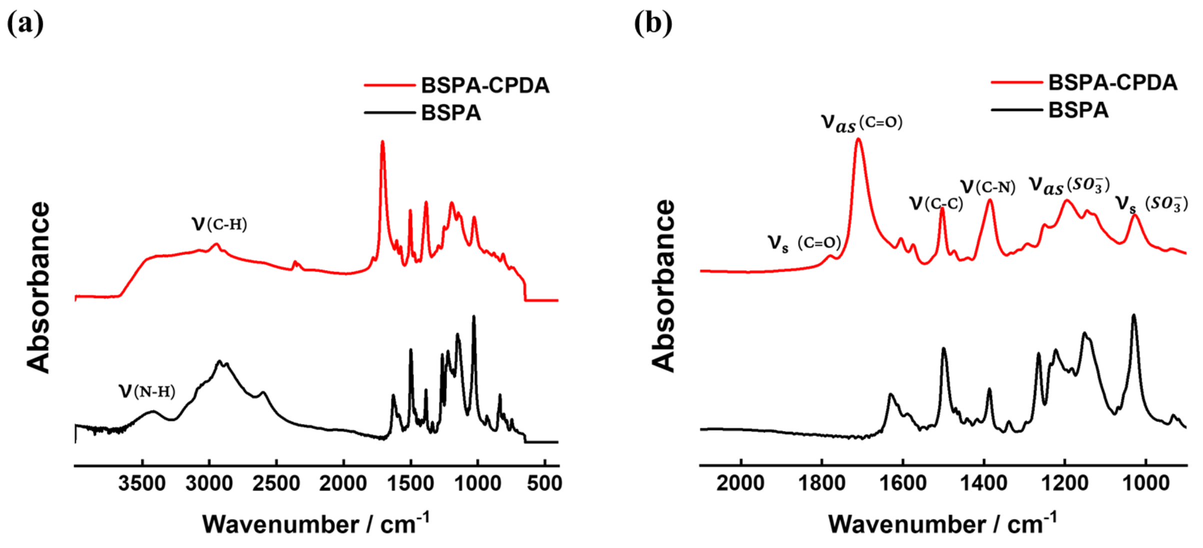

Figure S2. FTIR spectra of BSPA-CPDA (a) for 4000–400 cm^{-1} and (b) enlarge for the range of 2000–800 cm^{-1}.

3. Gel permeation chromatography (GPC) study

The molecular weight of BSPA-CPDA was measured by using gel permeation chromatography (GPC, LC-2000plus (JASCO)). The preparation process of the mobile phase solution of GPC is described as follows. The $NaNO_3$ (Kanto Chemical Co. Inc) (10.26 g) was dissolved in a mixed solvent of 600 ml of water and 400 ml of N, N'-dimethylformamide (DMF) (Fujifilm Wako Pure Chemical Corporation). Subsequently, 18 mL of acetic acid was added to the solution. Two columns Asahipak GF-7M HQ (Shodex) were used. The molecular weight of BSPA-CPDA was calculated from the calibration curve based on the standard materials (sodium polystyrene sulfonate, M_w = 505100, 126700, 16000 and 1690).

Figure S3 shows the GPC chromatogram of BSPA-CPDA. The calculation results of molecular weight were shown in Table S1. The number average molecular weight (M_n) and weight average molecular weight (M_w) were 4300 and 9300, respectively. Since no other prominent peaks were observed, the oligomer was sufficiently synthesized.

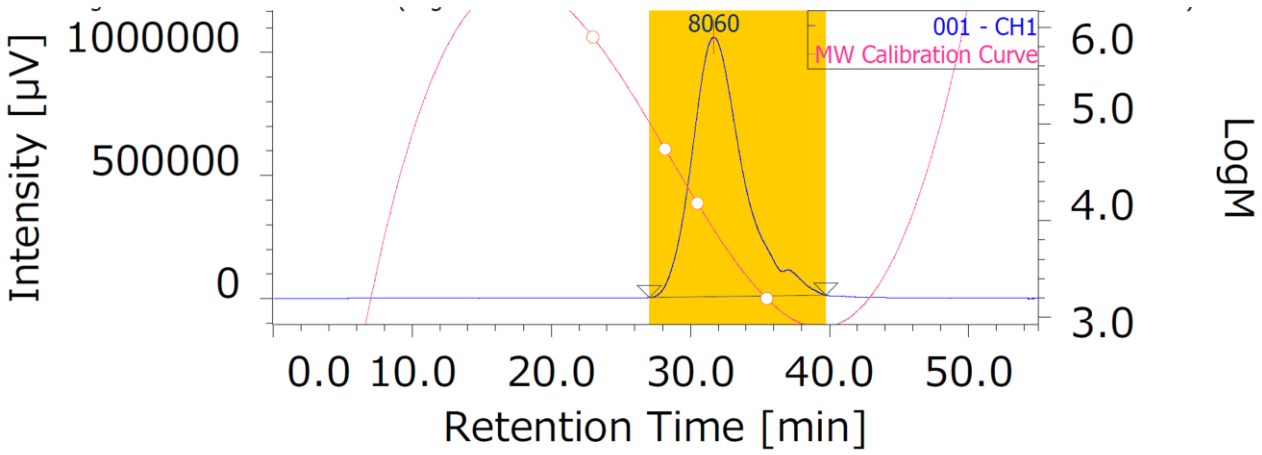

Figure S3. GPC spectra for BSPA-CPDA.

Table S1. Molecular weight of BSPA-CPDA.

	**Retention Time / min**	M_w	M_n	M_w/M_n
**BSPA-CPDA**	27.02 – 39.73	9365	4319	2.17

4. Chemical structure of ASSPI, ASPI-2 and ASPI-1

For comparison, Figure S4a and Figure S4b show the chemical structure of ASSPI (consisting of 1,2,4,5-cyclohexanetetracarboxylic dianhydride and BSPA)[S1], ASPI-2 (consisting of pyromellitic dianhydride and BSPA),[S2] and ASPI-1 (consisting of 1,4,5,8-naphthalenetetracarboxylic dianhydride and BSPA),[S3] respectively.

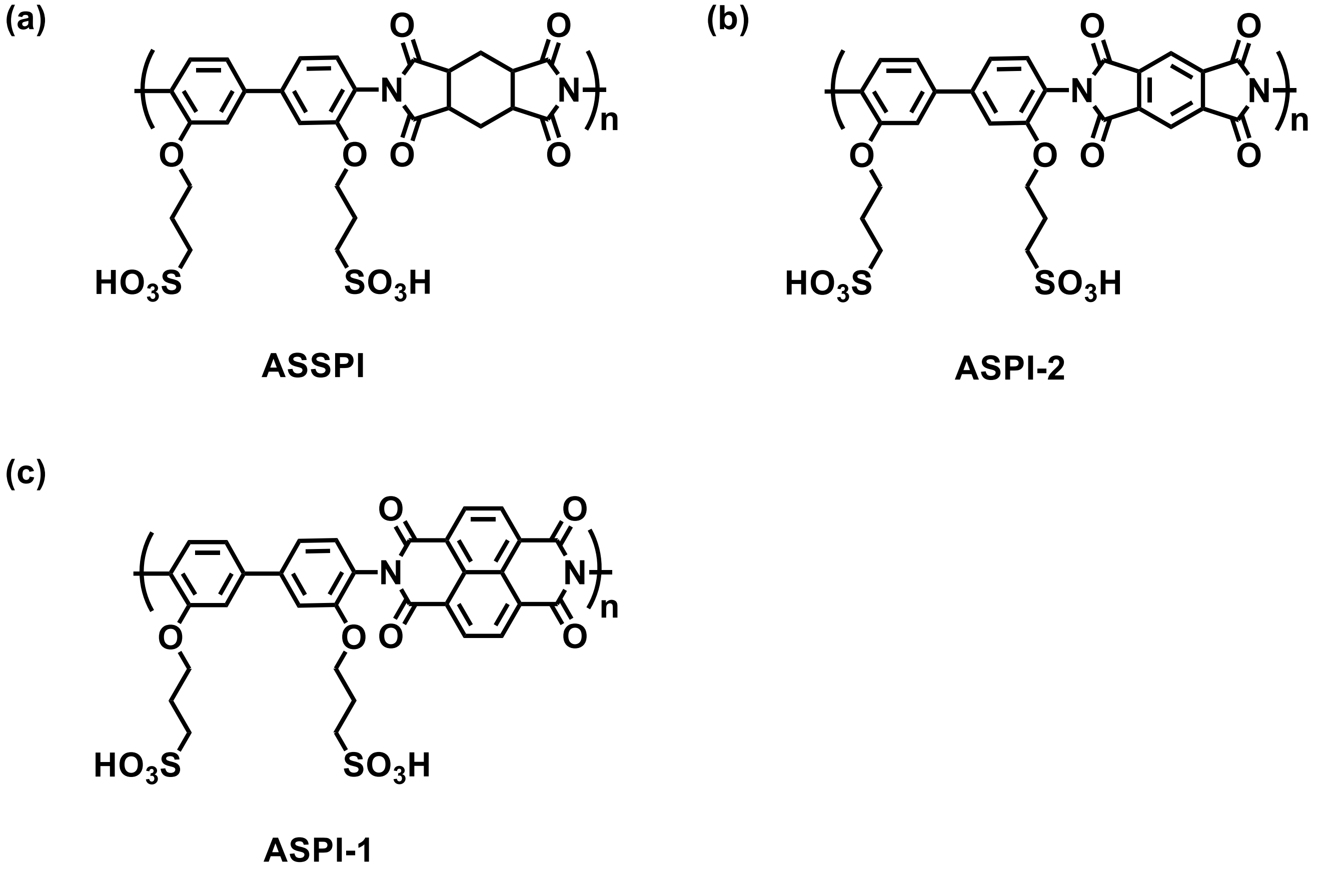

Figure S4. chemical structure of (a) ASSPI, (b) ASPI-2 and (c) ASPI-1.

5. Polarized Optical Microscopy (POM)

The POM images were taken by an optical microscope (BX51, BX51-P; Olympus Corp.) with a digital camera (DP28 camera; Olympus Corp.) All POM results were conducted at room temperature and ambient humidity.

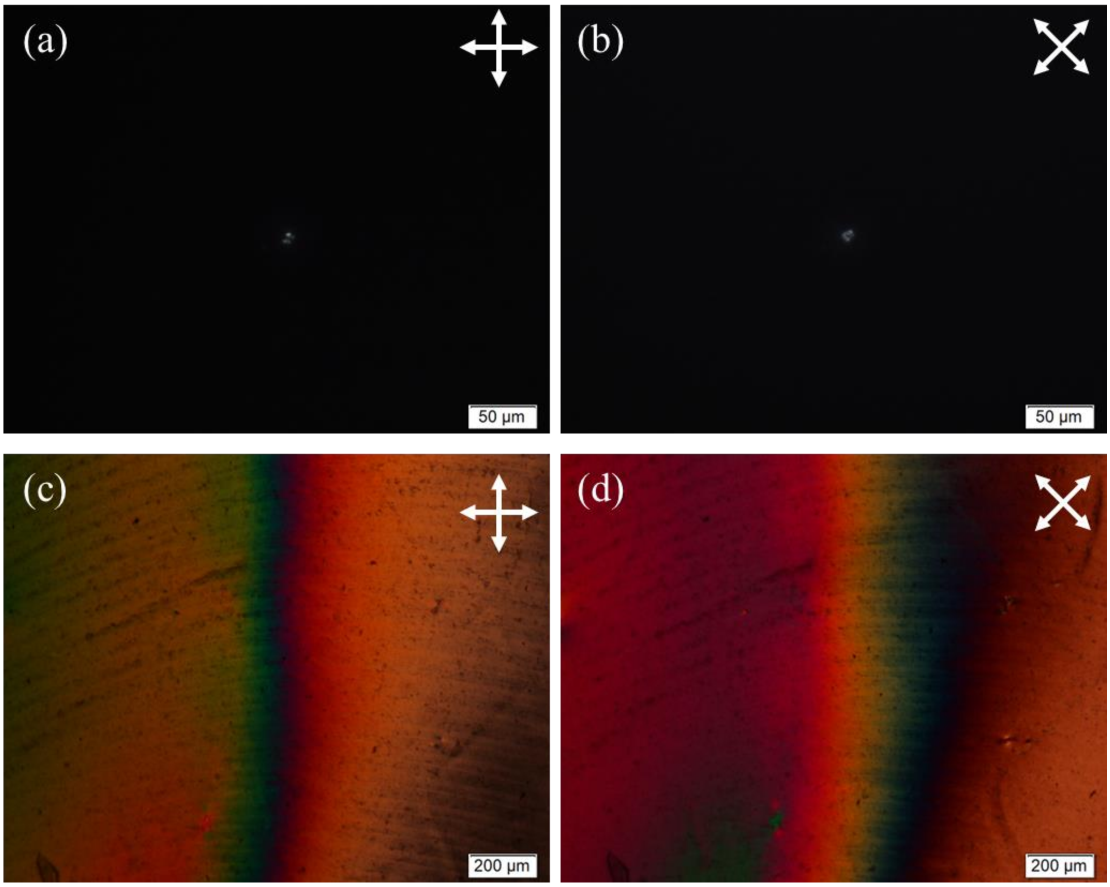

Figure S5. Polarized optical microscope images of the BSPA-CPDA (a), (b) thin film (500 nm thickness) and (c), (d) membrane (13 μm thickness).

References

(S1) Takakura, K.; Ono, Y.; Suetsugu, K.; Hara, M.; Nagano, S.; Abe, T.; Nagao, Y. Lyotropic Ordering for High Proton Conductivity in Sulfonated Semialiphatic Polyimide Thin Films. *Polym. J.* **2019**, *51* (1), 31–39. https://doi.org/10.1038/s41428-018-0111-1.

(S2) Krishnan, K.; Iwatsuki, H.; Hara, M.; Nagano, S.; Nagao, Y. Influence of Molecular Weight on Molecular Ordering and Proton Transport in Organized Sulfonated Polyimide Thin Films. *J. Phys. Chem. C* **2015**, *119* (38), 21767–21774. https://doi.org/10.1021/acs.jpcc.5b03292.

(S3) Ono, Y.; Goto, R.; Hara, M.; Nagano, S.; Abe, T.; Nagao, Y. High Proton Conduction of Organized Sulfonated Polyimide Thin Films with Planar and Bent Backbones. *Macromolecules* **2018**, *51* (9), 3351–3359. https://doi.org/10.1021/acs.macromol.8b00301.
